# Pediatric Nasal Tip Reconstruction After a Donkey Bite Using an Expanded Paramedian Forehead Flap With Conchal Cartilage Grafts: A Case Report

**DOI:** 10.7759/cureus.112005

**Published:** 2026-07-03

**Authors:** Mohammed Benlili, Ismail Hailouma, Doha Arreyouchi, Oufkir Ayat Allah

**Affiliations:** 1 Department of Plastic and Reconstructive Surgery, Faculty of Medicine and Pharmacy of Oujda, Mohammed First University, Oujda, MAR

**Keywords:** donkey bite, full-thickness nasal defect, paramedian forehead flap, pediatric nasal reconstruction, tissue expansion

## Abstract

Pediatric nasal reconstruction is challenging because it requires restoration of the external skin cover, structural support, and internal lining while taking into account facial growth and long-term aesthetic outcomes. Animal bite injuries may further complicate reconstruction because they often cause contaminated, irregular, composite tissue loss. We report the case of an eight-year-old boy who presented one year after a donkey bite with a 4 × 2 cm full-thickness nasal tip defect involving the medial portions of both alae, the anterior columella, and the anterior septal region. The child had loss of nasal tip projection, alar retraction, exertional nasal obstruction, and psychosocial discomfort. Reconstruction was performed in stages using a right paramedian forehead tissue expander, bilateral vestibular hinge flaps for internal lining, conchal cartilage grafts for alar support and columellar projection, and an expanded paramedian forehead flap for external skin coverage. Pedicle division was performed 25 days after flap transfer. The postoperative course was uneventful. At six months of follow-up, nasal breathing was normal, nasal tip projection was satisfactory, and no stenosis or secondary revision was required. A pressure-related frontal bony imprint caused by the tissue expander was noted intraoperatively and showed marked spontaneous regression after expander removal. This case highlights the value of a staged expanded paramedian forehead flap in selected pediatric patients with full-thickness nasal defects and limited forehead laxity. It also emphasizes the importance of reconstructing all three nasal layers and discussing the possibility of pressure-related cranial remodeling with families before pediatric forehead expansion.

## Introduction

Pediatric nasal reconstruction remains particularly challenging because of the three-dimensional anatomy of the nose, the need to restore the external cover, structural support, and internal lining, and the potential impact of reconstruction on facial growth and long-term aesthetic outcomes [1,2]. These difficulties are further amplified in posttraumatic defects caused by animal bites, which are often contaminated, irregular, and composite in nature, involving the skin, cartilage, and mucosa [3].

The forehead flap remains one of the most reliable options for reconstruction of large or full-thickness nasal defects because it provides a robust vascular supply and a good color and texture match [2,3]. In children, however, limited forehead laxity may compromise flap design and donor-site closure. Tissue expansion may therefore be particularly useful in selected cases, allowing recruitment of additional forehead skin, reducing donor-site tension, and improving contouring of the reconstructed nose [4].

We report the case of an eight-year-old boy presenting with a delayed full-thickness nasal tip defect after a donkey bite, reconstructed using a staged approach with an expanded paramedian forehead flap, bilateral hinge flaps for internal lining, and conchal cartilage grafts for structural support. The originality of this case lies in the unusual donkey bite etiology, the delayed presentation one year after injury, the need for tissue expansion because of limited forehead laxity, and the observation of a pressure-related frontal bony imprint after tissue expansion.

## Case presentation

An eight-year-old boy with no significant past medical or surgical history was referred to our department one year after sustaining a donkey bite injury to the nasal tip. Initial management at the time of injury consisted of wound irrigation, antibiotic therapy, and local wound care until complete healing was achieved. Tetanus and rabies vaccinations had been administered. However, the exact antibiotic regimen used during the early postinjury period was not available from the initial records, and precise information regarding early cartilage exposure or local signs of infection was not documented.

**Figure 1:**
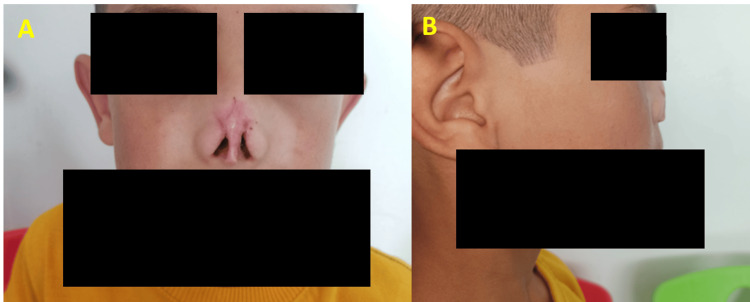
Preoperative clinical photographs (A) Frontal view showing a full-thickness nasal tip defect involving the medial portions of both alae, the anterior columella, and the anterior septal region. (B) Lateral view showing loss of nasal tip projection.

On clinical examination, the patient presented with a 4 × 2 cm full-thickness posttraumatic nasal defect involving the nasal tip, the medial third of both nasal alae, the anterior half of the columella, and the anterior part of the nasal septum. The surrounding skin was scarred, with retraction of the residual alar tissues and vestibular mucosa. This resulted in loss of nasal tip projection on profile view (Figure 1). Functionally, the patient complained of nasal obstruction during exertion, especially on the left side, secondary to tissue retraction. Functional nasal assessment was based on subjective symptom reporting and clinical examination. External examination showed alar retraction with narrowing of the affected nasal inlet. No formal instrumental assessment of nasal airflow was performed. The deformity also had a significant psychological impact on the child.

Forehead examination revealed tight skin with poor laxity. The distance between the eyebrow and the frontal hairline on the right side was approximately 6 cm. Because the defect involved the three essential layers of nasal reconstruction, such as skin cover, cartilaginous support, and internal lining, reconstruction with a skin graft alone was considered unsuitable. A staged reconstruction using an expanded right paramedian forehead flap was discussed with the parents. This option was selected because it could provide adequate skin coverage despite limited forehead laxity, improve donor-site closure, and allow simultaneous reconstruction of the nasal lining and framework. Written informed consent was obtained from the patient’s legal guardians for surgery and for publication of the clinical photographs.

First surgical stage: tissue expander placement

**Figure 2:**
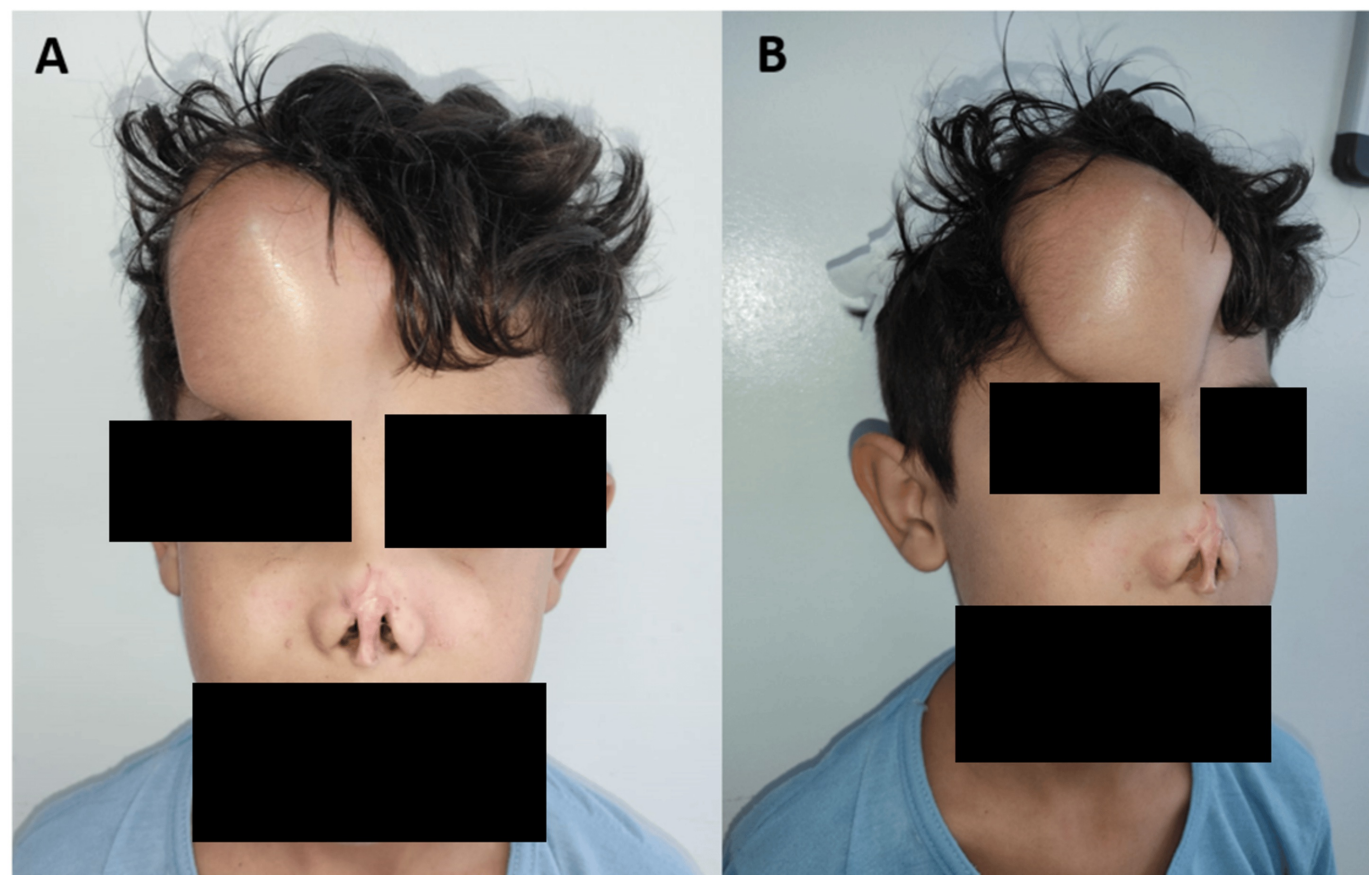
Appearance after forehead tissue expansion (A) Frontal view showing expansion of the right paramedian forehead skin. (B) Oblique view showing adequate expanded forehead tissue available for nasal reconstruction.

Thirteen months after the injury, a 100-cc rectangular tissue expander was inserted into the right paramedian forehead region in the subgaleal plane through a scalp incision. The subgaleal plane was selected to provide a stable pocket with adequate soft-tissue coverage over the expander. The valve was also positioned within the scalp. Perioperative antibiotic prophylaxis was administered. Expansion was started 15 days after expander placement and performed progressively. The injected volume varied between 5 and 9 cc per session, depending on the patient’s tolerance, with a final total volume of 97 cc. This gradual expansion protocol was used to avoid excessive tension and reduce pressure-related complications. Expansion was uneventful, with no infection, skin necrosis, or expander exposure. Adequate forehead skin was obtained for nasal resurfacing (Figure 2).

Second surgical stage: nasal reconstruction

After three and a half months, sufficient forehead skin had been obtained, and the second stage of reconstruction was performed. Perioperative antibiotic prophylaxis was also administered during definitive reconstruction.

The scarred edges of the nasal defect were refreshed, particularly at the level of the alar remnants. The internal lining was reconstructed using bilateral hinge flaps elevated from the vestibular walls. The anterior septal area was opened, and the hinge flaps were sutured to the septum using 5-0 Monocryl. Mobilization of the residual septal mucosa was also performed to contribute to the restoration of the internal nasal lining.

**Figure 3:**
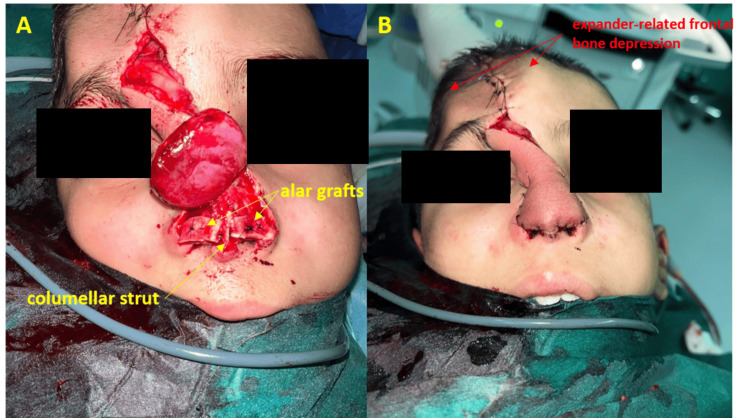
Intraoperative views of nasal reconstruction (A) Placement of conchal cartilage grafts; yellow arrows indicate the bilateral alar grafts and the columellar strut. (B) Intraoperative view after elevation of the expanded paramedian forehead flap; red arrows indicate the expander-related frontal bone depression.

Reconstruction of the cartilaginous framework was then performed using a conchal cartilage graft harvested from the right ear through a retroauricular approach. The graft was sculpted into two alar grafts and one columellar strut. The aim of cartilage reconstruction was to restore nasal tip projection, provide alar support, and prevent secondary stenosis (Figure 3A). The auricular donor site was closed, and a fatty gauze dressing was applied.

For external skin coverage, a right paramedian expanded forehead flap measuring 6 cm in width and 6.5 cm in height was designed according to a template matching the defect. During flap elevation, the superior portion of the expander capsule was excised, while the capsule around the pedicle was preserved. At that time, a pressure-related frontal bony imprint corresponding to the expander was noted (Figure 3B). The donor site was closed over a suction drain. Nasal packing and splints were placed at the end of the procedure.

The postoperative course was uneventful, with no infection, venous congestion, wound dehiscence, or flap necrosis.

Third surgical stage: pedicle division

Pedicle division was performed 25 days after flap transfer following a successful pedicle-clamping test, under sedation. The flap was divided and inset with contour refinement.

Follow-up and outcomes

**Figure 4:**
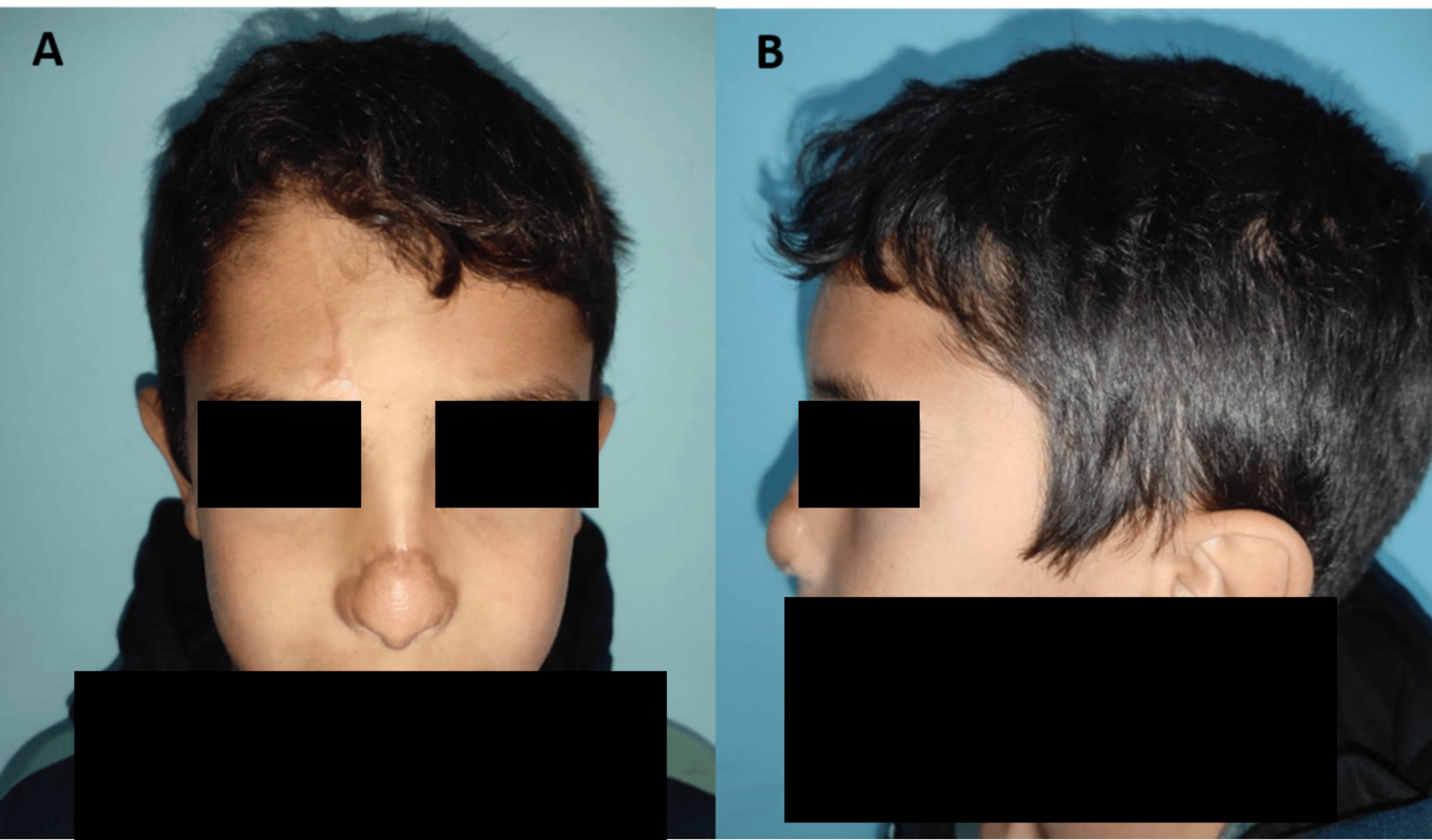
Postoperative result at six months after reconstruction (A) Frontal view showing restoration of nasal tip contour and projection, with a thin forehead scar. (B) Lateral view showing satisfactory nasal projection and improved nasal profile.

At six months of follow-up, the patient had regained satisfactory nasal tip projection (Figure 4). The airway was patent, with normal nasal breathing and no evidence of nasal stenosis. The forehead scar was thin but still mildly erythematous. The child and his parents were satisfied with the aesthetic and functional outcome, and social reintegration had improved.

Although no formal psychological scale was used, the parents reported improved self-confidence and social interaction after reconstruction. No secondary revision was required during the follow-up period.

Regarding the frontal bony imprint caused by tissue expansion, marked spontaneous regression was observed after expander removal. At six months, only a small residual external contour irregularity persisted.

The overall sequence of management from the day of injury to the last follow-up is summarized in Table 1.

**Table 1:** Timeline of management from the day of injury

Step	Timing from Day 0	Details
Donkey bite injury	Day 0	Nasal tip trauma with complex full-thickness tissue loss
Initial management	Day 0	Wound irrigation, antibiotic therapy, and local wound care
Vaccination	Day 0/initial management period	Tetanus and rabies vaccinations administered
Complete healing	During the months following the injury	Progressive healing with residual scar deformity and nasal retraction
Referral for reconstruction	Day 0 + 12 months	Evaluation of a complex full-thickness nasal defect
Tissue expander placement	Day 0 + 13 months	A 100-cc rectangular tissue expander was placed in the right paramedian forehead in the subgaleal plane
Start of expansion	Day 0 + 13 months + 15 days	Progressive expansion was initiated
Expansion period	From Day 0 + 13 months + 15 days to approximately Day 0 + 16.5 months	Expansion was performed with 5-9 cc per session, for a total injected volume of 97 cc
Definitive reconstruction	Approximately Day 0 + 16.5 months	Internal lining reconstruction, conchal cartilage grafting, and transfer of an expanded paramedian forehead flap
Pedicle division	Approximately Day 0 + 17.3 months	Performed 25 days after flap transfer following a successful pedicle-clamping test
Final follow-up	Approximately Day 0 + 23.3 months	Six-month follow-up showing good nasal tip projection, normal breathing, no stenosis, and no need for secondary revision

## Discussion

This case illustrates several key principles of pediatric nasal reconstruction. First, the defect was not a simple cutaneous loss. It was a 4 × 2 cm full-thickness defect involving the nasal tip, the medial third of both alae, the anterior columella, and the anterior septal region, with associated mucosal loss, alar retraction, loss of nasal tip projection, and exertional nasal obstruction. In such a setting, reconstruction with a skin graft alone would have been inadequate because it would not restore the three essential layers of the nose: internal lining, cartilaginous support, and external skin cover [1].

The subunit principle described by Burget and Menick remains highly relevant in this context [1]. The nasal tip and columella are central aesthetic subunits, and defects affecting these regions require reconstruction that restores contour, projection, and harmonious junctions with adjacent units. In our patient, the addition of bilateral hinge flaps for lining and conchal cartilage grafts for the framework was essential to achieve not only resurfacing but also structural and functional restoration. The cartilage grafts were particularly important for restoring nasal tip projection, supporting the alar margin, and reducing the risk of secondary stenosis.

The forehead flap has been shown to be a reliable option in children. In their series of children younger than 10 years, Giugliano et al. reported favorable outcomes with forehead flap reconstruction and emphasized that pediatric age alone should not preclude its use when indicated [2]. In our case, the main issue was the lack of forehead laxity, which made a conventional forehead flap less favorable. This justified the use of tissue expansion. Expanded forehead flaps have been described as a useful modification for larger or more complex nasal defects because they increase the amount of available tissue and facilitate donor-site closure [3,4]. In addition, expanded skin may provide a thinner and more pliable flap, which is advantageous for nasal contouring.

Our case also shares similarities with previously reported bite-related nasal reconstructions while remaining distinct in several respects. Hesamirostami and Hesamirostami reported reconstruction of a pediatric dog-bite defect involving the tip and columella using an expanded forehead scalping flap [4]. Menick reported extensive experience in nasal reconstruction using the three-stage forehead flap, highlighting its reliability for complex nasal defects requiring careful restoration of contour and structural support [5]. Compared with previously reported pediatric nasal bite reconstructions, the present case is notable for the unusual etiology of a donkey bite, the delayed presentation one year after injury, the use of a staged expanded paramedian forehead flap in a child with limited forehead laxity, and the observation of pressure-related frontal bony remodeling after tissue expansion.

Tissue expansion in children remains a valuable reconstructive tool, but it is associated with a recognized complication profile. Braun et al. summarized common complications of pediatric tissue expansion, including infection, implant exposure, extrusion, hematoma, device failure, and skin necrosis [6]. In the pediatric craniofacial region, pressure-related remodeling of the underlying skull is another important consideration. El-Saadi and Nasr demonstrated that tissue expansion in children can induce transient bony changes, including depression beneath the expander, with subsequent remodeling after expander removal in many cases [7]. Broader reviews have further emphasized the need for careful monitoring and management of complications during pediatric tissue expansion [8]. In our patient, a frontal bony imprint was observed intraoperatively after expander removal and showed marked spontaneous regression by six months, leaving only a small residual contour irregularity. This finding is clinically important because it should be discussed preoperatively with the family when forehead expansion is planned in a growing child.

More recently, Ryu and Jang reported forehead flap reconstruction for a dog-bite nasal injury with satisfactory functional and aesthetic outcomes [9]. Compared with that report, the present case further highlights the feasibility of staged expanded forehead flap reconstruction in a child with limited forehead laxity and a complex three-layer nasal defect.

Functionally and aesthetically, the postoperative outcome was satisfactory. The child regained satisfactory nasal tip projection, nasal breathing normalized, there was no residual stenosis, and no secondary revision was required during the six-month follow-up. The parents and the child were satisfied with the outcome, and social reintegration improved. This case supports the use of staged expanded forehead flap reconstruction in selected children with complex nasal defects when local tissue is insufficient and structural support is required.

The main limitation of this report is the relatively short follow-up period. Because nasal growth continues throughout childhood and adolescence, longer follow-up is planned to evaluate the long-term stability of nasal projection, airway patency, scar maturation, symmetry, and the possible need for secondary revision as the child grows. In addition, psychological improvement was assessed subjectively through parental reporting rather than with a validated quality-of-life or psychosocial assessment scale.

## Conclusions

Complex pediatric nasal defects after animal bites require reconstruction of all three anatomical layers of the nose: internal lining, cartilaginous framework, and external skin cover. In selected children with limited forehead laxity, a staged expanded paramedian forehead flap can provide reliable tissue for short-term resurfacing while facilitating donor-site closure. In our case, combining lining reconstruction, conchal cartilage grafting, and an expanded forehead flap achieved satisfactory short-term functional and aesthetic outcomes. A pressure-related frontal bony imprint should be recognized as a potential complication of pediatric tissue expansion. Longer follow-up is needed to assess nasal growth, long-term airway stability, scar maturation, and the possible need for secondary revision.
